# Metabolites Re-programming and Physiological Changes Induced in *Scenedesmus regularis* under Nitrate Treatment

**DOI:** 10.1038/s41598-018-27894-0

**Published:** 2018-06-27

**Authors:** Nyuk-Ling Ma, Ahmad Aziz, Kit-Yinn Teh, Su Shiung Lam, Thye-San Cha

**Affiliations:** 1 School of Fundamental Science, Universiti Malaysia Terengganu, Kuala Terengganu, Malaysia; 2 Institute of Marine Biotechnology, Universiti Malaysia Terengganu, Kuala Terengganu, Malaysia; 3 Eastern Corridor Renewable Energy Group (ECRE), School of Ocean Engineering, University Malaysia Terengganu, 21030 Kuala Terengganu, Malaysia

## Abstract

Nitrate is required to maintain the growth and metabolism of plant and animals. Nevertheless, in excess amount such as polluted water, its concentration can be harmful to living organisms such as microalgae. Recently, studies on microalgae response towards nutrient fluctuation are usually limited to lipid accumulation for the production of biofuels, disregarding the other potential of microalgae to be used in wastewater treatments and as source of important metabolites. Our study therefore captures the need to investigate overall metabolite changes via NMR spectroscopy approach coupled with multivariate data to understand the complex molecular process under high (4X) and low (1/4X) concentrations of nitrate (${{\bf{NO}}}_{{\bf{3}}}^{{\boldsymbol{-}}}$). NMR spectra with the aid of chemometric analysis revealed contrasting metabolites makeup under abundance and limited nitrate treatment. By using NMR technique, 43 types of metabolites and 8 types of fatty acid chains were detected. Nevertheless, only 20 key changes were observed and 16 were down regulated in limited nitrate condition. This paper has demonstrated the feasibility of NMR-based metabolomics approach to study the physiological impact of changing environment such as pollution to the implications for growth and productivity of microalgae population.

## Introduction

Nitrogen is a primary nutrient regulating growth and metabolism in microalgae, often absorbed in the form of nitrate. Nitrogen is important mainly for tissue growth and protein synthesize^1^. The ability of microalgae to adapt to varying levels of macronutrients and micronutrients in the environment are proven of an extensive mechanism to holistically reallocate its metabolic components in order to create a response and sustain through the adverse situations^2,3^.

Although response toward differing levels of nutrients are species-specific, subsequent studies have shown that minor manipulation onto the medium’s nutrient content will induce physiological and biochemical changes in carbohydrate, protein and lipid production. Microalgae adapt to varying levels of nutrients via two strategies; luxury consumption (direct uptake in excess without immediate need) and energy mitigating for nutrient supply (directing use of carbon reserves during starvation periods)^4,5^. However, exactly how microalgae adapt to one of these two strategies is not fully understood yet. Abundance of nitrate may activate luxury uptake and results in the accumulation of amino acid precursors (NO_3_^−^ and NH_4_^+^) and nitrate reductase^3,4^. Nitrogen is either assimilated into holding within their cell wall or converted into nitrogen-based reserves such as simple amino acids^4,6^. Recent molecular studies carried out on *Chlamydomonas reinhardtii* found a family of three protein transporters NRT1 (NPF), NRT2, and NAR1 are responsible for determining substrate specificity and affinity of nitrate uptake in regards to nitrate concentration^7^.

Physiologically, nitrate limitation triggers a stress response in microalgae and are often associated with slow growth rates, decreased production in amino acids and declining enzymatic activity leading to reduce photosynthetic capability^3,4,8^. Respiratory metabolism becomes secondary as more energy is focussed on reallocation of metabolites designed for storage such as nonphosphorylated polyglucans and lipids^9^.

More notably, nitrogen limitation has been studied for its triacylglycerol (TAG)-enhancing properties and possible formation of economically important secondary metabolites. Many of the microalgae studied reported ~20–40% of increase in total lipid content and a shift towards production of neutral storage fatty acids such as TAG^1,6,10–12^. Changes in metabolic activities in nitrate and phosphate deplete mediums are also associated with production of reactive oxidative species (ROS) which are believed to trigger the assimilating of nutrients and energy into storage lipids^13^. This is because storage lipids and TAG synthesis provides an electron sink for excess electrons that occur during photo-oxidative stress thus serving as outlets to detoxify the lipid membrane^14,15^. Production of secondary carotenoid metabolites (asthaxantin, β-carotene, lutein) are associated with the TAG pathway hence some microalgae like *Dunaliella sp*. and *Hematococcus sp*. are able to produce these economically important antioxidants under nutrient stress^12,14^. Nevertheless, the responses toward nutrient stress is species specific, eg *Chlorella sorokiniana* and *Chlorella minutissima* showed only a minor increase in lipid accumulation^16^, while huge decline of antioxidant activities was detected in *Phaeodactylum tricornutum* and *Chlorella vulgaris*^12^. Therefore, in order to capture the metabolic changes on a wider scale, an analytical method that enables examination of individual metabolite developments rather than total extract changes is needed. Nuclear magnetic resonance (NMR) is a reliable and unbiased spectroscopy technique that can capture what happens metabolically on a wide scale^15^. Acquisition time for samples is relatively short and hence is time-saving compared to other analytical methods^17^. NMR coupled with chemomeric analysis is a powerful tool for discriminating between groups of related samples and identifying regions of spectrum that can be dedicated to further analysis. Nutrient limitation and abundance brings out a myriad of reactions that is of great benefit if explored to its full potential. By knowing the cellular changes under different culture conditions, the full potential use of microalgae for different usage, eg pharmaceutical, bioremediation, or as biofuel production can be explored. By using *Scenedesmus regularis* or synonym of *Pectinodesmus regularis*^18^ as microalgae model, this study demonstrated some important biochemical developments and changes in appearance, biomass accumulation, chlorophyll content and metabolites reprogramming when exposed to the presence of different nitrate (N) concentration.

## Results and Discussion

### Biomass and related growth

**Figure 1:**
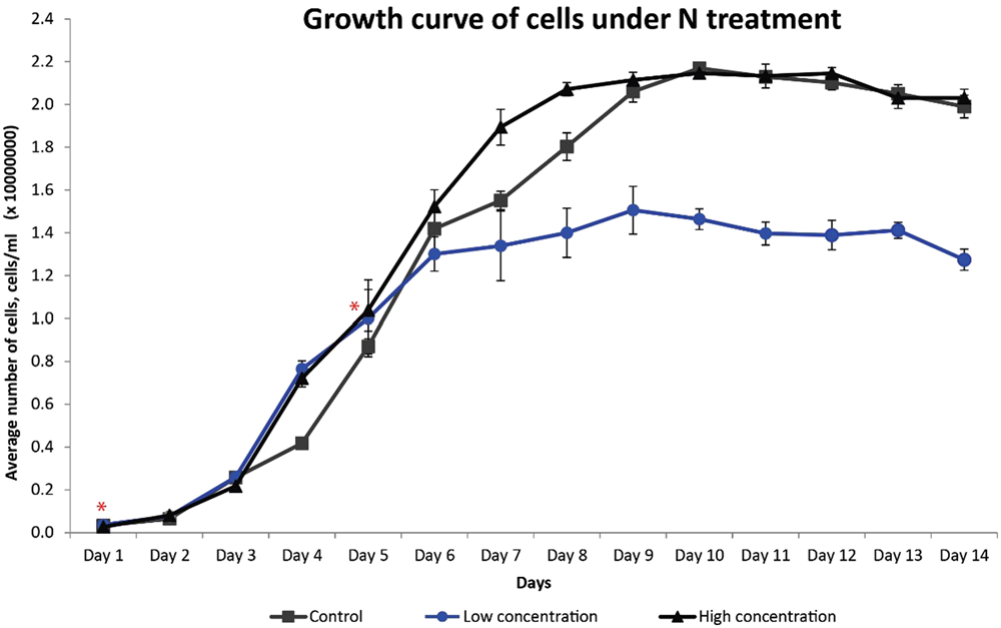
Growth curve of *S. regularis* under different concentrations of NO_3_ treatment. Points show average concentration of cells in 1.6 L of liquid culture from each batch of culture. The difference in exponential growth phase of 1/4X NO_3_ with the control and 4X NO_3_ treatment is noted with asterisks (*).

Algal biomass gained from each treatment was evaluated in order to understand how growth was affected. Approximately one fold decrease in mass gain was observed in nitrate depleting culture when compared against 4X and control NO_3_ treatment. The reduction of biomass was correlated with the reduction of growth rate observed in 14 days of treatment (Fig. 1). In nitrate depleting environment, *S. regularis* was found enter stationary phase mush earlier than in other treatment starting from day 6 (Fig. 1). Nitrate is the most important nutrient depended upon at the initial stage of growth in microalgae thus a lack of NO_3_ stunted microalgal growth^7^. Besides primary growth, nitrate is utilised as building blocks of genetic materials (DNA and RNA), proteins for cell expansion and pigment building blocks^1,3^. Therefore, it is not surprise that under limited nitrate condition, when the nitrate was used up after one week of culture, most of the cell division stop and push *S. regularis* moved into stationary growth phase. Contrarily, when additional of NO_3_ was available, carbon fixation occurs and prolong the exponential growth phase, which was in agreement of batch photobioreactors of *Scenedesmus* and *Chlorella* cultures^19^. Biomass accumulation of the control and 4X treatments in both nutrients did not differ significantly might due to the level of NO_3_ abundance were probably not enough to trigger a sudden “bloom” in its numbers as it has been reported that levels of NO_3_ have to be over 4.0 mg/L^20^. Rapidly utilisation of nitrate but not causing bloom phenomenon in high nitrate culture condition may suggest an application in wastewater treatment^21^. Wastewater from industrial, agricultural or urban can serve as growth substrate to support microalgae biomass production^22^.

### Chlorophyll content and total carotenoids

**Figure 2:**
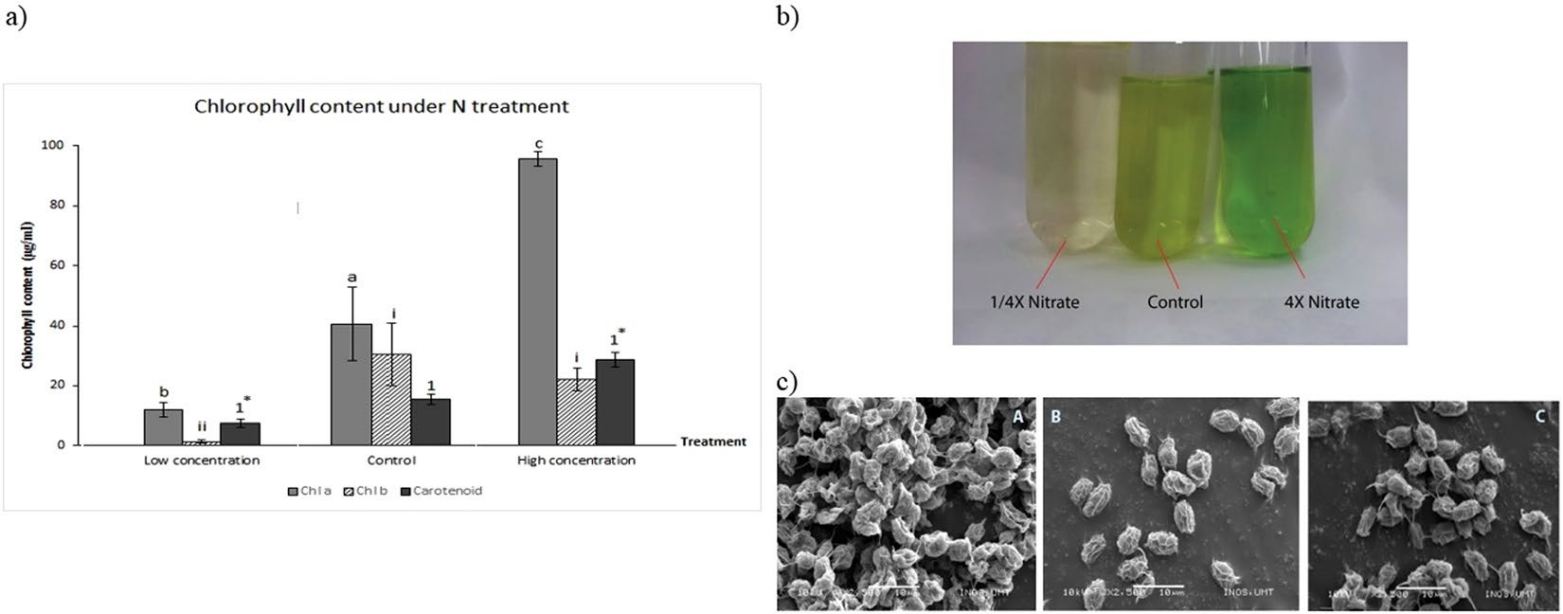
(**a**) Chlorophyll and carotenoid content of *S. regularis* measured from cultures under NO_3_ treatment. Alphabets, roman numerals and numbers represent significant different between chl a, chl b and carotenoid respectively. Asterisks (*) mark significant difference between treatments group. (**b**) Comparison of colour in medium culture of 1/4X NO_3_, control and 4X NO_3_. (**c**) Scanning electron microscope (SEM) images of *S. regularis;* (A) 1/4X NO_3_, (B) control and (C) 4X NO_3_.

Chlorophyll pigments are important light harvesting proteins that ultimately vary in concentration under the microalgae’s environment conditions. There are evidences shown that nitrate is an important building block for chlorophyll^12,13^. Chlorophyll pigments in cultures of limited NO_3_ were drastically reduced in comparison to control and high NO_3_ cultures (Fig. 2a). Due to very low chlorophyll content, the microalgae in nitrate-limited treatment appear pale green with hints of yellow (Fig. 2b). This condition known as chlorosis or “bleaching” is characteristic of NO_3_ limitation^12,13,23^. Chloroplasts serve as large reserves of nitrogen sources and has been shown to be degraded in a contractual autophagy mechanism in times of nitrogen starvation so that the remnants of degraded chlorophylls may be recycled to support metabolic needs that require NO_3_^3,4,14,24,25^. The highest chlorophyll content was achieved under 4X NO_3_ treatment with total chlorophyll a and b measured at 107.2 µg/ml. Chl a accumulation was often observed coinciding with high rates of cell division^23^, similar finding were observed in this study where the highest biomass produced in 4X nitrate treatment. The high Chl content would also coincide with higher photosynthesis activities and production of reactive oxygen species (ROS) as photosynthesis by products. Nevertheless, plants equipped with carotenoids that play photoprotective roles in the photosystem^12^. As pointed out by Singh, *et al*.^14^, nutrient stress activates TAG-related secondary carotenoid formation and can be seen as a way to combat high ROS activity that happens during stressed conditions. Therefore, higher Chl a levels coincided with carotenoid levels that were averagely higher than the control in 4X NO_3_ treatment. While under limited nitrate condition, photo-oxidative stress occurs with ROS attacking the chlorophyll system but plants fail to trigger more carotenoids to overcome the effect of oxidative stress which would explain no significant increase after being cultured in NO_3_ limited conditions^12,13^. Under limited NO_3_ treatment, cells appeared to be clumped together, shrivelled in appearance and spine formation were observed (Fig. 2c). According to^26^, the length of the spines represent the nitrate starvation experienced in microalgae. Interestingly, cells cultured in nitrate abundant cultures did not produce any signs of clumping and were observed to be more prone to form unicellular cells instead of paired sets of coenobia.

### Metabolites detected from aqueous solvent

**Figure 3:**
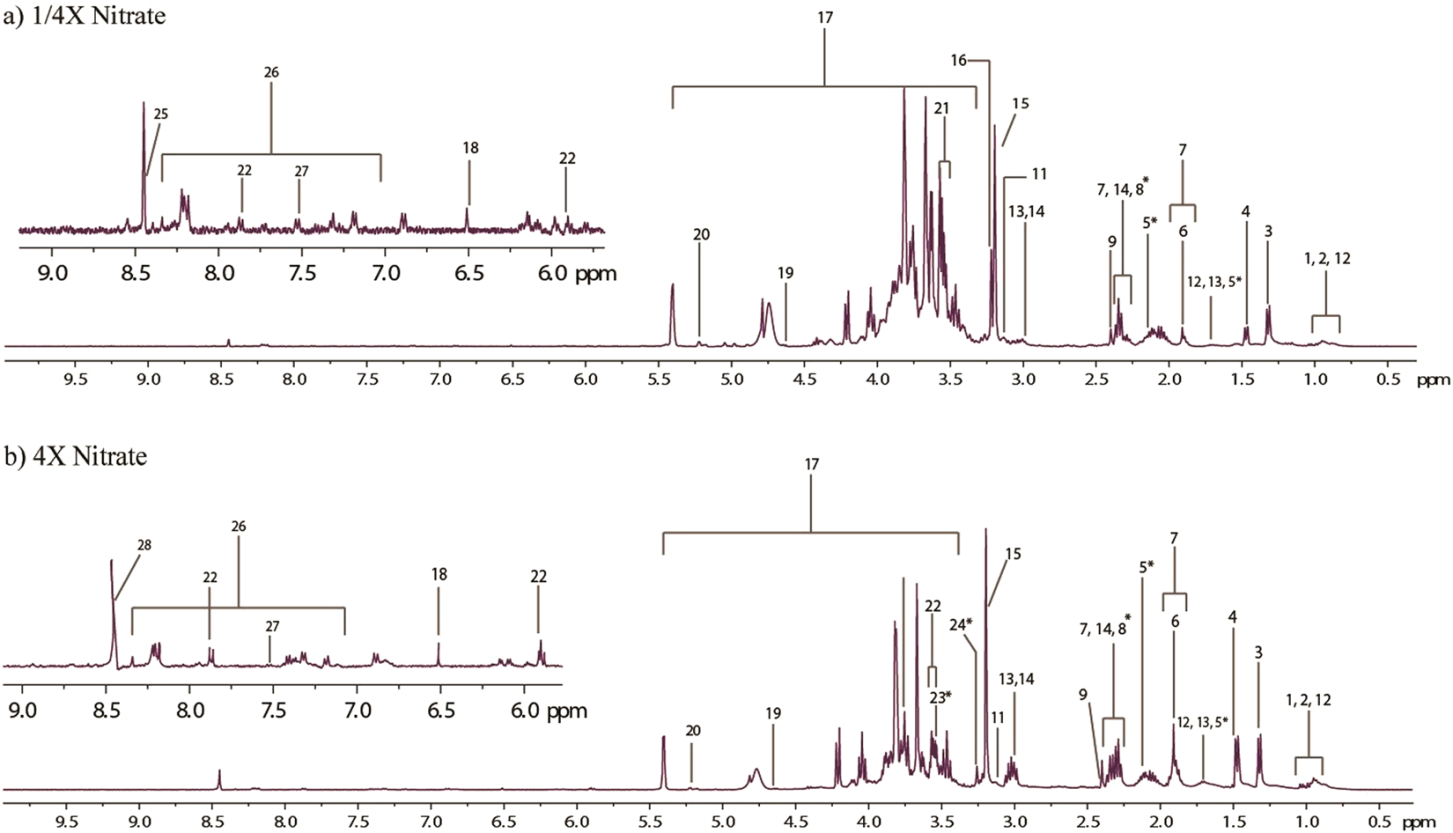
1H NMR spectra of *S. regularis* extracted using aqueous solvent from (**a**) 1/4X Nitrate and (**b**) 4X Nitrate treatment. Metabolites marked with an asterisk appear only in the treatment sample and not in control. Key for spectra refer to Table 1.

^1^H-NMR spectroscopy carried out on aqueous extracts returned rich spectra. Figure 3 shows a representative 400 Mhz NMR spectra of a microalgae sample from aqueous extracts of treated sample. The spectra was utilised for the identification of several endogenous metabolites (Table 1). Comparing spectra data from aqueous extracts of treated samples (Fig. 3a and b), some new peaks (trimethylamine, betaine and glycine) were observed in comparison to the control sample. This indicates an elicited response for certain metabolites when exposed to nutrient fluctuations. Choline-2 produces a prominent singlet at 3.19 ppm and is related to resonance from free choline while Choline-1 singlet at 3.20 ppm is a culmination of glycerophosphocholine, phosphorylcholine and minute influences from free choline^27^. Choline production is an aliphatic monoamine commonly partitioned metabolically as phospholipid- related compounds with the most common being phosphotidylcholine (PC) in microalgae. Ethanolamine produced a visible peak only in NO_3_ abundant cultures. Ethanolamine is often related as precursors of betaine-type lipids besides being incorporated as phosphotidylethanolamine (PE) into cell membranes^28^. Betaine lipids are naturally occurring ether-linked glycerolipids containing a betaine moiety with no phosphate group. Betaine was detected in control and NO_3_ abundance treatment but was absent in NO_3_ limited cultures (Fig. 3a). The structural capacity of cell membranes in microalgae typically contain diacylglyceryl-3-O-4′-(N,N,N-trimethyl)- homoserine (DGTS), a betaine lipid and PC in interchangeable amounts. It has however, been observed by Boroda, *et al*.^29^ that the cell membrane constitution does not distinguish between PC or DGTS as both lipids probably have equivalent functions. Trimethylamine is a constituent for the production of betaine lipids and can be correlated to an increase in ethanolamine production. Glycerol moiety was detected at δ 3.55. Glycerol is an important constituent in regards to both neutral and phospholipid-type lipids and is procured from the direct glycerol pathway or from the fermentation of starch^14,30^.

**Table 1:** Proton chemical shifts observed in *Scenedesmus regularis* treated with different nitrate concentration (a) metabolite and (b) fatty acid fraction.

Metabolites	H Group	δH^+^ (Multiplicity)	Solvent
Lipoproteins	CH_3_	0.91 (m)	Aqueous
Leucine	Terminal-CH_3,_ γ-CH_3_	0.95 (t), 1.72 (m)	Aqueous
Valine	Terminal CH_3_, Terminal CH_3_, β-CH_3_, α-CH_3_	0.98 (d), 1.03 (d), 2.2 (m), 3.63 (d)	Aqueous
Lactate	CH_3_, CH	1.33 (d), 4.1 (q)	Aqueous
Alanine	CH_3_, CH	1.48 (d), 3.76 (qt)	Aqueous
Lysine	γ-CH_2_, δ-CH_2_, β-CH_2_, ɛ-CH_2_	1.5 (m), 1.72 (m), 1.9 (m), 3.03 (t)	Aqueous
Spermine	H8, H3, (H2, H9, H6, H11)	1.71 (m), 2.12 (m), 3.14 (m)	Aqueous
Acetic acid	CH_3_	1.91 (s)	Aqueous
Gamma-amino-N-butyrate	β-CH_2_, α-CH_2_,	1.91 (q), 2.31 (t)	Aqueous
Butyric acid-4-amino		1.9 (m), 2.3 (t), 3.03 (t)	Aqueous
Glutamate	β-CH_2_, γ-CH_2_, α-CH	2.1 (m), 2.33 (dt), 3.77 (t)	Aqueous
Succinate	2xCH_2_	2.40 (s)	Aqueous
Ethanolamine	CH_2_NH_2_, CH_2_OH	3.14 (s), 3.85 (s)	Aqueous
Choline-1	CH_3_,	3.19 (s)	Aqueous
Choline-2	CH_3_, CH_2_NH	3.20 (s), 3.55 (m)	Aqueous
Betaine	CH_3_, CH_2_	3.26 (s), 3.91 (s)	Aqueous
Sucrose	H10; H9; OH; H17,19; H5, H3, H7	3.46 (t), 3.54 (d), 3.66 (s), 3.81 (m), 4.05 (t), 4.21 (d), 5.4 (d)	Aqueous
Glycine	CH	3.55 (s)	Aqueous
Glycerol	CHOH, CHOH, COH	3.55 (d), 3.65 (d), 3.77 (m)	Aqueous
α-glucose	CH-1, CH-6	3.81 (m), 5.22 (d)	Aqueous
β-glucose	CH-1	4.63 (d)	Aqueous
Uracil	H5, H6	5.8 (d), 7.52 (d)	Aqueous
Uridine	H(a), H-6	5.89 (t), 7.86 (d)	Aqueous
Fumarate	CH	6.51 (s)	Aqueous
Nucleoside/Nucleoside		7.1–7.9	Aqueous
Formate		8.45 (s)	Aqueous
Phosphatidylcholine	N(CH_3_)_3_, CH_2_OP	3.22 (m), 4.27 (m)	Chloroform
Glyceroglycolipids	CH_3_-sn3	4.05 (m)	Chloroform
Phosphatidylglycerol	Glycerol moiety	3.61 (m)	Chloroform
Phospholipid		3.1–3.9	Chloroform
Phosphatidylserine	CH_2_OP	4.28 (m)	Chloroform
Diacylglycerophospholipid	CH_3_-sn3	4.05 (m)	Chloroform
Triglyceride	CH sn1, 3(a), CH sn1, 3(b), CH sn2	4.31 (m), 4.14 (m), 5.26 (m)	Chloroform
Diglyceride	CH sn1, CH sn2	4.31 (m), 5.13 (m)	Chloroform
Squalene	4xCH_3_, 2x terminal CH_3_,=CHCH_2_	1.61(d), 1.69 (d), 2.04(m)	Chloroform
Sterol	CH_3_–18	0.53 (m)	Chloroform
Carotenoids	C=CH, CH=CH, CH-C(CH_3_)	6.14 (m), 6.64 (m), 6.35 (m)	Chloroform
Phaeophytin	N-H (pophyrin ring)	−1.41 (s), −1.61 (s)	Chloroform
Olefinic protons (alkene)	CH=CH/−C=CH	5.8–6.8	Chloroform
Aldehyde	CH=O	8.0 (m), 8.55 (m), 9.40 (m), 9.5 (m), 9.53 (m), 10.4 (m)	Chloroform
Ester	CH_2_C=O	2.32 (triplet of doublets)	Chloroform
Free fatty acid	CH_2_C=O	2.35 (doublet of singlet)	Chloroform
PUFA	Bis-allylic chains	i) C 18:2–2.77 (m) ii) C 18:3–2.81(m) iii) C 22:6–2.84(m)	Chloroform
**b) Fatty Acid Chains**			
Methyl end	n-CH_3_	0.88 (m)	Chloroform
PUFA methyl end	n-CH_3_	0.98 (m)	Chloroform
Alkyl chain	(-CH_2_-)_n_	1.25 (m)	Chloroform
Carboxylic end	CH_2_COO^_^	1.61 (m)	Chloroform
Alkyl chain (allylic)	CH_2_CH=CH…CH=CHCH_2_	2.06 (m)	Chloroform
Carboxylic end^*^	CH_2_COO^_^	2.34 (m)	Chloroform
Alkyl chain (bisallylic)	-CH=CH(CH_2_CH=CH)_n_	2.81 (m)	Chloroform
Double bonds	-CH=CH-	5.4 (m)	Chloroform
PUFA methyl end	n-CH_3_	0.98 (m)	Chloroform
Alkyl chain	(-CH_2_-)_n_	1.25 (m)	Chloroform
Double bonds	-CH=CH-	5.4 (m)	Chloroform

Amine based metabolites are nitrogenous compounds and it is not surprising to observe the reduction of amine and amino acids compounds in limited nitrate condition. Chlorophytes usually contain mono, di- and polyamines in varying amounts. These often contribute either to odour, toxins or play ecologically important roles within the microalgae^31^. Amines also exhibit certain biological activity and hold a potential for developing value-added products; spermine as an example involved in nucleic acid synthesis and cell division^31^. Choline, ethanolamine and trimethylamine are common amines often included into cell wall constitution to maintain fluidity and selectivity of the cell membrane.

Carbon source such as alpha- and beta-glucose were also observed in spectra (Fig. 3). Microalgae have adapted to storing carbon in the form of a disaccharide because it is more stable chemically thus allowing the cell to also use sucrose as an osmolyte^32^. Spectra obtained from 1/4X NO_3_ cultures were not as sharp as spectra obtained from other treatment cultures. Broad spectra are indicative of heavy molecular weight components inside the extract sample^15,33,34^. A possible higher relative concentration of sugar or starch granules (in comparison to other metabolites) may have accumulated within the microalgae cell as microalgae cells have been acknowledged to synthesis starch under nitrate starved conditions^8,35,36^.

Simple osmolytes (sucrose δ 3.66, betaine δ 3.25 and glycine δ 3.55) identified from the spectra show different intensity peaks among treatments. Two intermediates of the tricarboxylic-cycle (TCA) were also detected from all spectra. Fumarate produced a single singlet at δ 6.51 while succinate produced a singlet at δ 2.40 which can interconvert and produce succinyl-CoA, another TCA intermediate. Glutamate, TCA intermediate, was also detected across all spectra. The TCA cycle is therefore indicative of an aerobic route used by *S. regularis* for procuring its energy currency and subsequent metabolites. Furthermore, metabolites indicative of fermentative pathways were also identified (lactate at δ 1.33; formate at δ 8.45; acetic acid at 1.91).

### Metabolites detected from chloroform solvent

**Figure 4:**
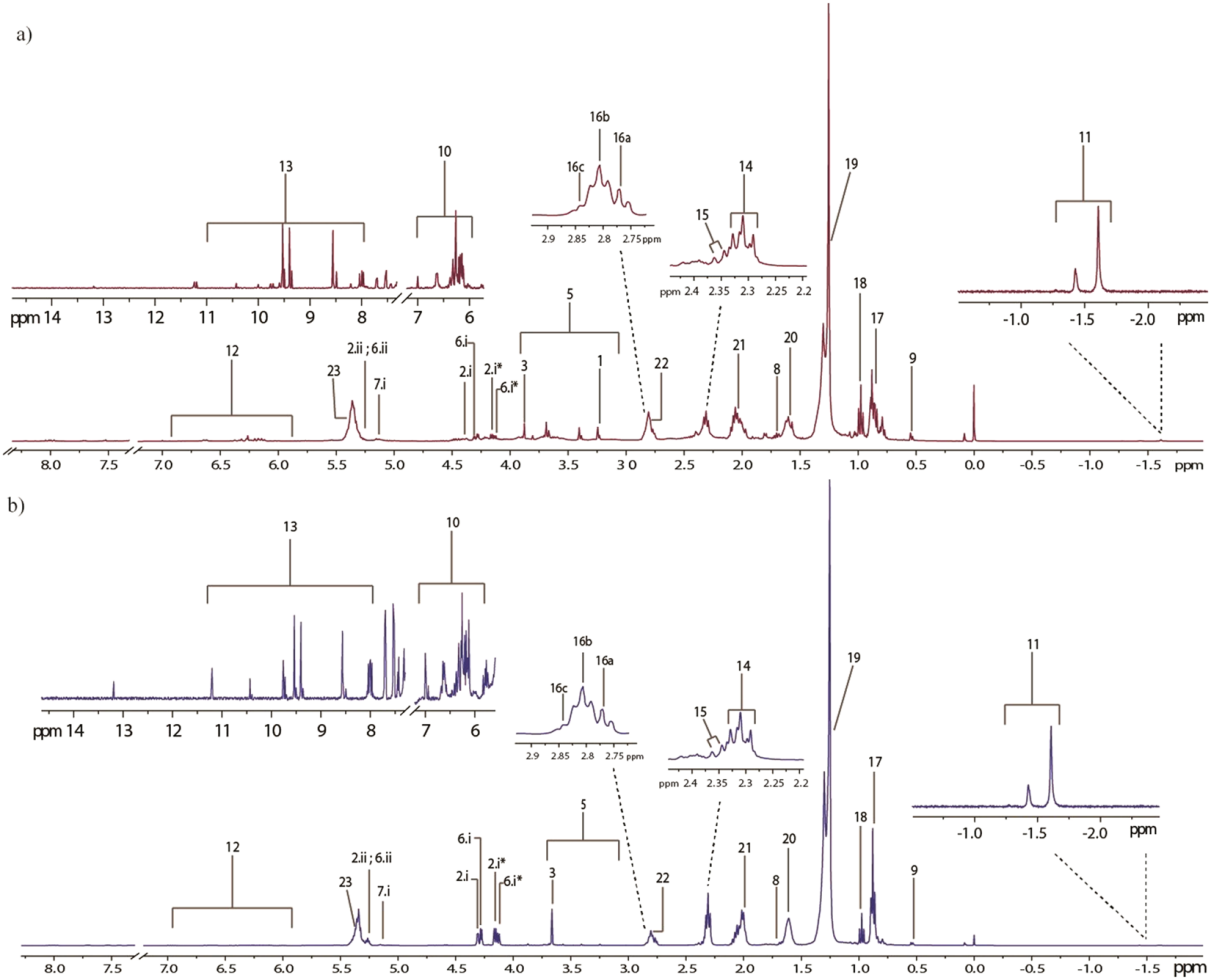
1H NMR spectra of *S. regularis* extracted using chloroform solvent from (**a**) 1/4X Nitrate and (**b**) 4X Nitrate treatment. Metabolites marked with an asterisk appear only in the treatment sample and not in control. Key for spectra refer to Table 1.

Lipids accumulated by microalgae can be divided into polar and non-polar lipids (neutral) lipids (Fig. 4, Table 1). Both types of lipids follow very clear and distinct accumulation strategies and may transform from one to another depending on the needs of cells^37,38^.

Microalgae primarily store lipid since formation of lipid requires powerful reducing cofactors and ATP^39^. These lipids can then be used to either stabilize cell due to its hydrophobic nature or broken down to release energy when required. The signals produced by the protons linked on double bonds from polyunsaturated fatty acids (PUFA) culminate in a multiplet signal at δ 5.4. The allylic chain protons give rise to signals at δ 2.06 and δ 2.81 while protons located near the carboxylic end of the chain produce signals at δ 1.61 and δ 2.34. Protons situated along the long saturated carbon chain gives a very obvious multiplet at δ 1.25. It is interesting to note that fatty acid chains ending with a methyl- end attached with a double bond at an omega position gives rise to signals at δ 0.98 while non-omega methyl end protons culminate in a signal at δ 0.88. Recent studies have designated signals at the multiplet δ 2.81 to correspond to double bonds of three types of PUFA; docosahexaenoic acid, linoleic acid, α-linolenic acid^40^. The corresponding signals, marked 16a, b and c, were also observed in this study at: (i) δ 2.77 for linoleic acid (C 18:2); (ii) δ 2.81 for α-linolenic acid (C 18:3); (iii) δ 2.84 for Docosahexaenoic acid (C 22:6/DHA). Signals related to carotenoid were found in the region of δ 6.0–δ 6.7. According to Sobolev, *et al*.^41^, salient signals appearing in the region between δ 6.0–δ 6.7 can be attributed to a fragment of conjugated trans double bonds, represented chemically as –(CH_3_)-C=C**H**-C**H**-C**H**-C–(CH_3_)-, common to all carotenoids. The protons (in bold) give rise to signals of multiplets at δ 6.14, δ 6.64 and δ 6.35 in the literature. A structure related to chlorophyll, i.e. phaeophytin, produces two prominent singlets at δ −1.43 and δ −1.61 in a solvent of CDCL_3_^41^. Two prominent singlets were also observed at δ −1.41 and δ −1.61 in this study thus proving presence of chlorophyll-related molecules in the non-aqueous mixture. Carotenoids are important pigments of the photosynthetic apparatus and also act as powerful antioxidant to relief the cell from excess photo-oxidation^42^.

Methyl group of C-18 sterol structures of different sterols often resonate at different ppm. β-sitosterol for example, resonates at δ 0.68 while stigmasterol produces a signal at δ 0.7^40,41^. As a general rule, C-18 moiety of methyl groups belonging to sterol compounds resonates at δ 0.6–δ 0.8^41^. Our study furnished a signal at δ 0.53, pointing to presence of ergosterol-like sterols in the mixture. Microalgae may synthesis either glycerides or fatty alcohols as their main neutral lipid storage^43^. Triacylglycerides (TAG) are the major storage lipids in a majority of microalgae^44^. Spectral ID returns glycerol moieties detected at δ 4.31, δ 4.14 and δ 5.26.

Chemical shifts coinciding with signals related to phospholipids are usually detected in the region δ 3.0–δ 4.05^40,45^. Higher intensity signals (characteristic of phospholipid head group signals) were detected between the region of δ 3.1–δ 4.05 in nutrient abundant and control spectra in comparison to nutrient limited spectra suggesting reduced phospholipid production under nutrient limitation. Signals corresponding to phosphate head groups (CH_2_OP) connected to phosphatidylcholine and phosphatidylserine were also detected at δ 4.27 and δ 4.28 respectively. The phosphate head group signal at δ 4.02 corresponding to phosphatidylethnolamine^41,46^ however was not detected. Diacylglycerophospholipids (glyceroglycolipids) presented very weak signals at δ 4.05 and is almost not visible in nutrient limited spectra. This shows the flexibility of the microalgae’s metabolome to shift from membrane-lipid production to storage lipid and vice versa under different environmental cues.

Literature data show alkenes/fatty alcohols (olefinic protons) and aldehydes producing characteristic signals at δ 5.8–δ 6.8 and δ 8.0–δ 11.0 respectively^40^. These signals are a mixture of signals from fatty hydrocarbons and conjugated/non-conjugated protons of oxylipins of PUFAs such as DHA^40^. Fatty esters and free fatty acids were also detected in the spectra. In regards to their physiological roles, not much is known^47^. Some secondary fatty aldehydes and alkenes from microalgae have been studied for their antimicrobial properties and thus may have been synthesised by microalgae as an ecological defence system^48,49^.

### Multivariate analysis

**Figure 5:**
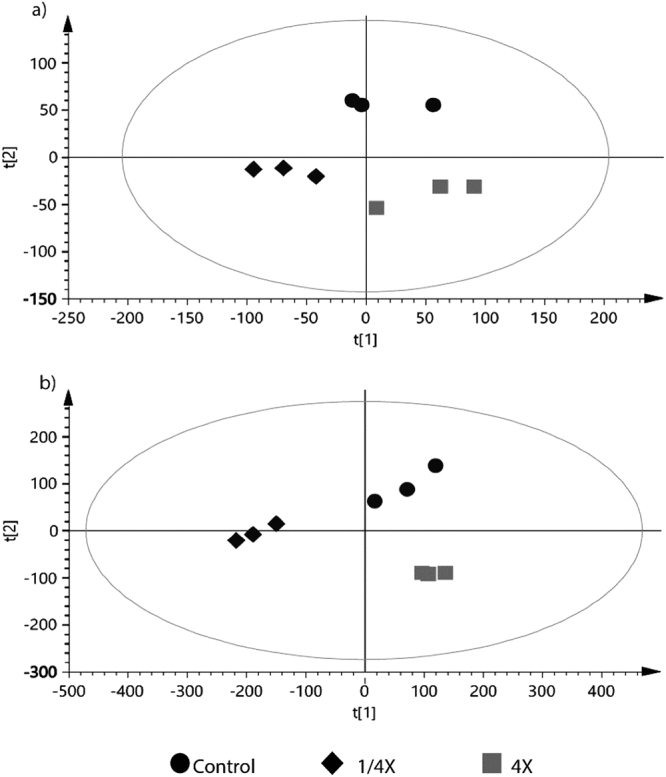
PLS-DA grouped according to different nitrate concentrations. (**a**) Metabolome extracted from aqueous (**b**) metabolome extracted from chloroform. Clear separation is observed among the different levels of nutrient treatment, eclipse on the plot represents the 95% confidence interval.

The supervised PLS-DA plot the variation that occurs by separating between groups observed and segregation of the studied group. Both aqueous and chloroform sample showed similar pattern, in which clear cluster were observed between three treatment (Fig. 5). Clustering among groups of treatments was more apparent in the chloroform extracts of nitrate (Fig. 5b; R^2^X = 0.896). Lower R^2^X values in aqueous extracts of nitrate treatment (Fig. 5a; R^2^X = 0.76), signifies a partial diversion in the degree of response towards different nutrient conditions. High predictability values from the PLS-DA score plots (>0.7) shows high reproducibility of data from treatments.

**Figure 6:**
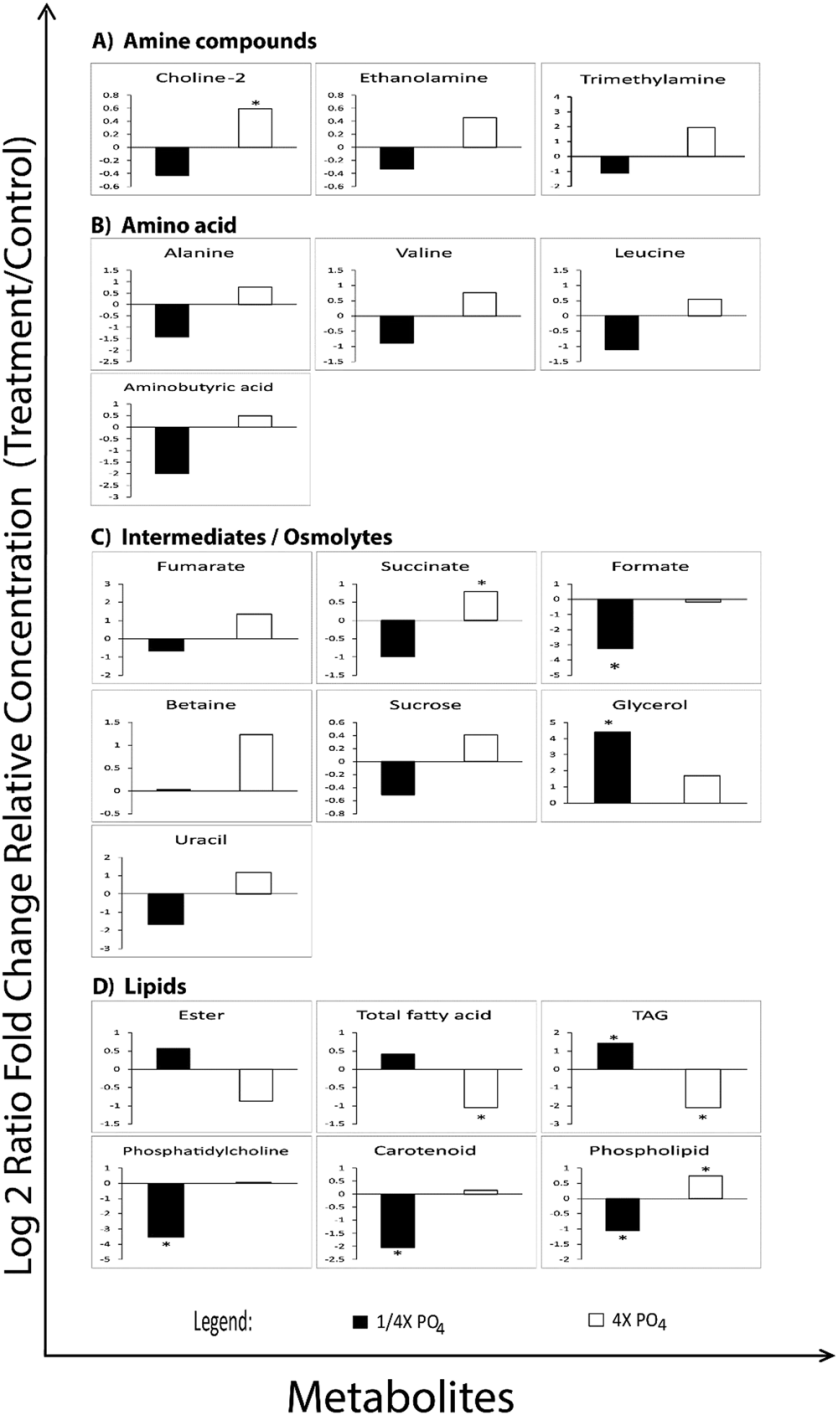
Fold changes of metabolites plotted against relative concentration of the control. Asterisks represents significant different between each nutrient treatment (*t-*test; *P* < 0.05).

**Figure 7:**
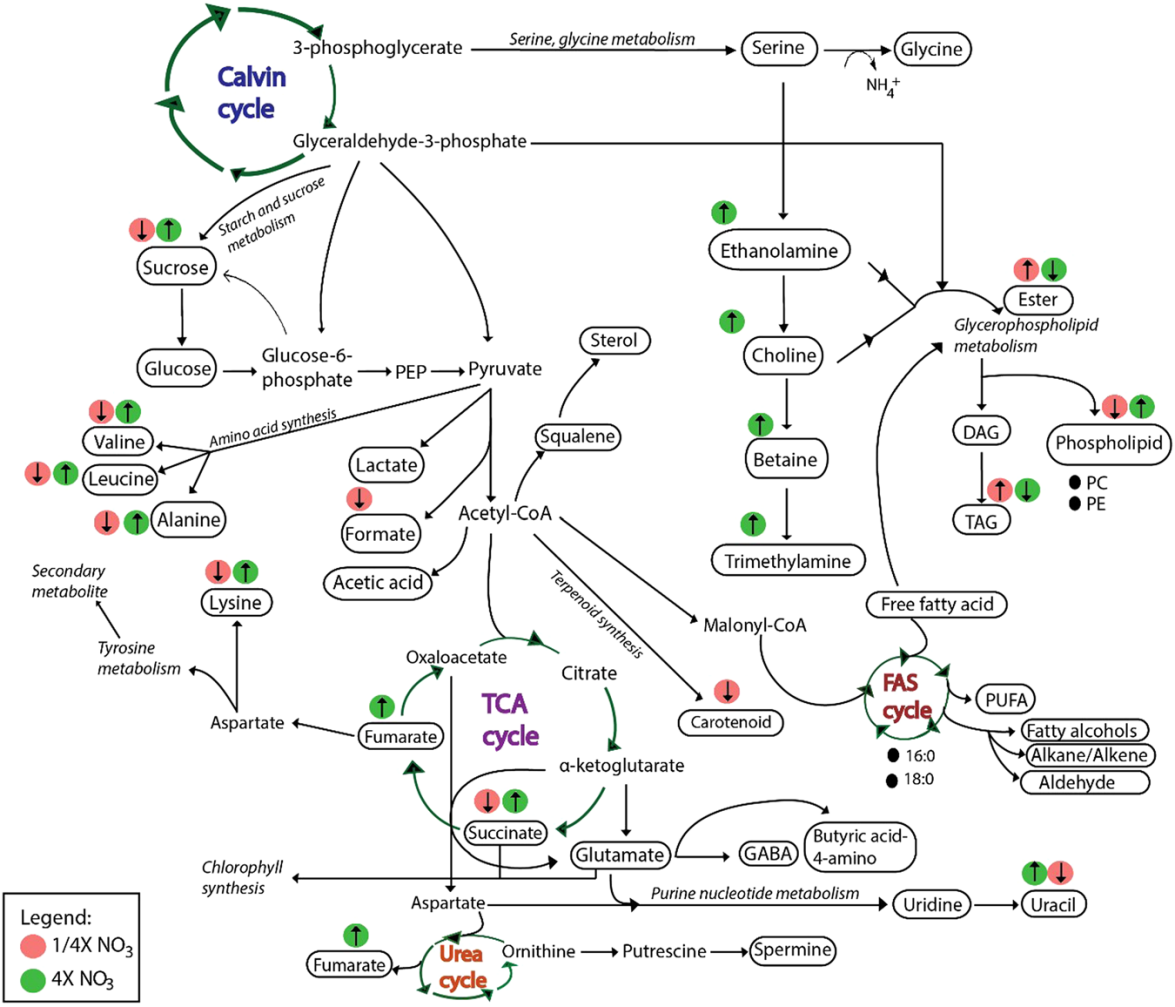
Metabolite regulation pathways involved in *S. regularis* under nitrate treatment. Key for abbreviations: (i) FAS: Fatty acid synthesis; (ii) PC: Phosphatidylcholine; (iii) PE: Phosphatidylethanolamine; (iv) PEP: Phosphoenulpyruvate; (v) TCA: Tricarboxylic cycle; (vi) TAG: Triacylglyceride; (vii) DAG: Diacylglyceride; (viii) GABA: γ-aminobutyric acid.

Following PLSDA analysis, the data represent each metabolites were all log2 transformed and tested with student T-test between treatments. Metabolite that show significant different p < 0.05 were then plotted as relative to control into bar charts (Fig. 6). Significant difference between treatments and control were observed in membrane-lipid related metabolites (Choline-2, ethanolamine, betaine), amine compounds and amino acids (trimethylamine, alanine, valine, leucine, aminobutyric acid), TCA cycle intermediates (succinate, fumarate, uracil), energy-related/osmolytes metabolites (sucrose, formate, glycerol) and lipid fragments (ester, fatty acids, TAG, phosphatidycholine, carotenoid and phospholipid) (Fig. 6). These metabolites are involved in Calvin cycle, TCA cycle, Urea cycle and Fatty acid synthesis cycle (Fig. 7). Fluctuations in aforementioned metabolites prove that different nutrient regimes are able to affect the up-regulation and down-regulation of several important pathways.

In 20 metabolites that detected significant versus to control, apart of lipids fragments, all were increased under 4X nitrate treatment, while mostly reduced its content under limit nitrate treatment. Amino acid synthesis pathways were most severely affected in low nitrate treatments. Amino acid contents (alanine, valine, leucine, aminobutyric acid) were significantly reduced in nitrate limited cultures. This in turn would explain that under nitrate limitation, the rate of protein synthesis was greatly reduced thus affecting the synthesis of chlorophyll proteins due to the low nitrate substrate to support synthesis of amino-containing compounds. Example is the reduction of chlorophyll content (Fig. 1) as a direct consequence of reduced chlorophyll protein under nitrate limitation, as the synthesis of chlorophyll requires 4 molecules of nitrogen. However, there seems to be another route of amino acid synthesis and catabolism adopted by microalgae by proteolysis of ribulose-1,5-bisphosphate carboxylase oxygenase (Rubisco) and conversion of other N-containing materials such as nucleotides and their derivatives to maintain amino acids synthesis^44^. The increment of amino acid and amine compounds were observed in nitrate abundance condition. This is in agreement with treatment of diatom in nitrogen replete treatment, where all amino acids were found increased except histidine and methionine^18^. Similar explanation for the succinate and fumarate which may be a direct consequence of increased nitrate availability thus providing the cell enough substrate to support photosynthesis, which increases energy output of the cells. This increased energy-production coincides with increased sucrose levels in 4X NO_3_ cultures. Luxury uptake of nitrate increases photosynthetic rate thus diverting the excess carbon into storage components within the cell.

Nitrate is an important content for the production of RNA material in cells^50^. Uracil, an amino containing nucleotide, was observed to be reduced considerably under nitrate limitation but significantly increased under nitrate abundance condition. Limitations on DNA/RNA material synthesis may inhibit cell growth or reduce cell division rates in nitrate limited microalgal cultures. Reduction of osmolytes or osmoprotectants may be a common response to nitrate limited stress^44^. Osmolytes accumulate in the cytoplasm and mainly play an active role in maintaining the ionic balance, help in scavenging ROS, regulating pH and for maintaining the osmotic pressure (osmoregulation) within the microalgae cell^43,51^. Production of osmolytes are dependent on environmental cues and may be synthesised at any stage of growth^51^. Betaine levels were significantly elevated in 4X NO_3_. Betaine may play dual roles as a non-phosphorus containing cell wall component or as an osmolyte in the microalgae cell^51^.

Glycerophospholipid pathways leading towards formation of phospholipids and pyrimidine biosynthesis pathways were found up-regulated in nitrate abundance. Contradictory, low nutrient condition stimulates phospholipid hydrolysis and the activation of acyl-hydrolase therefore increasing fatty acid content in the cell^52^. The cell switches its mode of synthesis from forming polar lipids to storage lipids. Increased in fatty acid and TAG was associated with degradation of phospholipids and phosphatidycholine, suggesting that TAG was recycled from membrane proteins^44^. Nevertheless, while nitrate limitation has been known to achieve higher lipid to protein ratio within the cell, behaviour of polar metabolites such as carbohydrates, proteins and phosphatases are not popularly known to be affected^3,53–56^. Alternatively, exogenous bioactive molecules and light intensity can be utilised in growth promotion and lipid content accumulation in microalgae^57,58^.

## Materials and Method

### Microalgae cultures and nitrate treatment

*Scenedesmus regularis* (KS-MC1) was obtained from Institute of Marine Biotechnology (IMB) of Universiti Malaysia Terengganu. The culture and maintenance of *Scenedesmus regularis* was adopted from^34^. In nitrate treatment, the concentration for NaNO_3_ was altered while the other components remained the same as in Guillard’s F2 media. The concentrations for NaNO_3_ in control cultures is 8.824 × 10^−1^ mM, in 4X nitrate treatment, concentration of NaNO_3_ was modified to 3.528 mM while in 1/4X treatment, NaNO_3_ was modified to 2.205 × 10^−1^ mM. An inoculum concentration of 1 × 10^5^ cells/ml was transferred to freshly prepared treatment media. The treatment cut off point is on Day 8 of culture. For each treatment, three biological samples were used and the experiment were repeated three time.

### Quantification of Chlorophyll Content

Chlorophyll content was extracted by taking 1.5 ml aliquots of culture media containing microalgae from each treatment and centrifuged at 10,000 rpm for 5 minutes to get 200 µl of supernatant. Pellet was rehydrated with 1 ml of methanol (Merk, ACS grade) and left to shake at 300 rpm on heating plate at 60 °C for 5 minutes. Experiments were carried out under dark conditions. Tubes were then removed from the heating plates and left to stand in the dark for 24 hours at 4 °C. The tubes were subsequently centrifuged for 5 minutes at 10 000 rpm and the supernatant were collected. Chlorophyll content was determined using a spectrophotometer and measured the absorbance at wavelength 665 and 652 nm^59^. Chlorophyll content calculated in µg/ml was determined according to Taylor and Fletcher^60^ formula as follows:

$${\rm{Chlorophyll}}\,{\rm{a}}:{c}_{a}(\mu g/ml)=16.72\,{A}_{665.2}-9.16\,{A}_{652.4}$$

$${\rm{Chlorophyll}}\,{\rm{b}}:{c}_{a}(\mu g/ml)=34.09\,{A}_{652.4}-15.28\,{A}_{665.2}$$

$${\rm{Total}}\,{\rm{carotenoid}}:{c}_{(x+c)}(\mu g/ml)=(1000\,{A}_{470}-1.63\,{c}_{a}-104.96{c}_{b})/221$$

### Biomass yield

The wet biomass were harvested from 1.2 L of liquid culture then frozen overnight at −80 °C before subjected to freeze drying (LABCONCO) at −80 °C until constant weight were obtained. The biomass yield was expressed as difference between final dried weight minus initial fresh weight.

### SEM preparation and analysis

Fresh microalgae samples in 50 ml was collected by centrifuged and the pellet was underwent general fixation overnight at room temperature using 2.5% glutaraldehyde in 0.1 M sodium cacodyle. Solution was then discarded and pellet rinsed with 0.1 M sodium cocadylate buffer (pH 7.2). Pellet was then submerged in 1% osmiumtetraoxide prepared in 0.1 M sodium cocadylate buffer and left to stand for 4 hours. The pellet was then rinsed with buffer and underwent a series of dehydration steps using various concentrations of ethanol. Pellet was then mounted on specimen stub and left to dry in a fume hood with the aid of hexamethyldisilazane (HMDS – Merk, ACS grade). Specimen stub was then coated with gold using auto fine coater (INOS, UMT).

### Extraction procedure and NMR spectra acquisition

A total of 0.215 g of freeze-dried algal biomass was used for (homogenizer) extraction^34^. Extraction solvents were prepared by adding Chloroform (99% absolute chloroform, ACS grade) with aqueous solvent that prior mixed from 99% absolute methanol (ACS, ISO, Reag grade) and pure distilled water into 1:1 ratio. Two layers of supernatant formed following each extraction and each layer was collected separately and dried up in freeze drier. The aqueous phase of extract was re-suspended in 600 µl of phosphate buffer (0.1 M, pH 7) containing 10% D_2_O and the chloroform phase extract was re-suspended in 600 µl of chloroform –d 99.8% containing 0.01% of sodium tetramethysilane (TMS). Both sample were then subjected to 10 min of 10,000 rpm centrifugation before transferred into 5 mm NMR tube^34^.

### NMR analysis and data reduction

Analysis and further furnishing of NMR peaks (baseline correction, phasing, solvent peak removal) were carried out in Mnova (Ver 9.0 – Mestrelab) and Topspin (Ver 3.2 – Bruker). TMS (δ 0.0) was used as the reference point in chloroform spectra while alanine (δ 1.48; doublet) was used as reference peaks in aqueous spectra. The interfering signal of suppressed H_2_O resonance and CHCl_3_ in aqueous and chloroform spectra was removed. NMR peaks were then identified based on their integration, chemical shifts and peak multiplicity.

### Multi-variant analysis

All variables were log-centred and scaled to unit variance before proceeding with supervised and unsupervised multivariated analysis, namely Principal Component Analysis (PCA) and Partial List Squares Discriminant Analysis (PLS-DA) using SIMCA (SIMCA-P + 1.3 − Umetrics) software. The validity of the models was assessed by statistical parameters R2 (correlation coefficient) and Q2 (cross-validation correlation coefficient). Average of each variable were first Log2 transformed and Two tailed student’s T-tests were carried out on metabolites to determined statistical differences between input variable and cut out point at p < 0.05.

## Conclusion

Cell cultures respond to nutrient fluctuations through a myriad of strategies. Under N deficiency, production of photosynthetic pigments is severely stunted. NO_3_ addition lead to chlorophyll pigments and amino acid accumulation while NO_3_ limitation had very low amino acid content but was very effective at increasing neutral lipid content. Multi-variate analysis showed that more changes were observable in non-polar extracts when microalgae are exposed to nutrient deficiency.

A basic fingerprint of the biochemical changes using NMR technology coupled with multi-variate analysis proved the capability of NMR as a time-saving and efficient data mining analytical tool as biochemical changes in both polar and non-polar could be captured simultaneously. More studies need to be carried out in order to determine the correct proportions of nutrient content in which to stimulate production of favourable metabolites without compromising productivity. Metabolite activity are important indicators in pathway alterations which would come in handy when determining biomarkers or designing nutrient regimes for economical output.
